# Identification of a novel *WAS* mutation in a South African patient presenting with atypical Wiskott-Aldrich syndrome: a case report

**DOI:** 10.1186/s12881-020-01054-6

**Published:** 2020-06-05

**Authors:** Brigitte Glanzmann, Marlo Möller, Mardelle Schoeman, Michael Urban, Paul D. van Helden, Lisa Frigati, Ravnit Grewal, Hermanus Pieters, Ben Loos, Eileen G. Hoal, Richard H. Glashoff, Helena Cornelissen, Helena Rabie, Monika M. Esser, Craig J. Kinnear

**Affiliations:** 1 DSI-NRF Centre of Excellence for Biomedical Tuberculosis Research, South African Medical Research Council Centre for Tuberculosis Research, Division of Molecular Biology and Human Genetics, Faculty of Medicine and Health Sciences, Stellenbosch University, P.O. Box 241, Cape Town, 8000 South Africa; 2 Division of Paediatric Infectious Diseases, Department of Paediatrics and Child Health, Tygerberg Children’s Hospital, Faculty of Medicine and Health Sciences, Stellenbosch University, Cape Town, South Africa; 3 Division of Haematology, National Health Laboratory Services, Tygerberg Hospital, Cape Town, South Africa; 4 Department of Physiological Sciences, University of Stellenbosch, Stellenbosch, South Africa; 5 Immunology Unit, Division of Medical Microbiology, National Health Laboratory Service and Faculty of Medicine and Health Sciences, Stellenbosch University, Tygerberg Hospital, Cape Town, South Africa; 6 Department of Haematopathology, National Health Laboratory Service and Faculty of Medicine and Health Sciences, Stellenbosch University, Tygerberg Hospital, Cape Town, South Africa

**Keywords:** Wiskott-Aldrich syndrome, Exome sequencing, Primary immunodeficiency diseases

## Abstract

**Background:**

The X-linked recessive primary immunodeficiency disease (PIDD) Wiskott-Aldrich syndrome (WAS) is identified by an extreme susceptibility to infections, eczema and thrombocytopenia with microplatelets. The syndrome, the result of mutations in the *WAS* gene which encodes the Wiskott-Aldrich protein (WASp), has wide clinical phenotype variation, ranging from classical WAS to X-linked thrombocytopaenia and X-linked neutropaenia. In many cases, the diagnosis of WAS in first affected males is delayed, because patients may not present with the classic signs and symptoms, which may intersect with other thrombocytopenia causes.

**Case presentation:**

Here, we describe a three-year-old HIV negative boy presenting with recurrent infections, skin rashes, features of autoimmunity and atopy. However, platelets were initially reported as normal in numbers and morphology as were baseline immune investigations. An older male sibling had died in infancy from suspected immunodeficiency. Uncertainty of diagnosis and suspected severe PIDD prompted urgent further molecular investigation. Whole exome sequencing identified *c. 397 G > A* as a novel hemizygous missense mutation located in exon 4 of *WAS*.

**Conclusion:**

With definitive molecular diagnosis, we could target treatment and offer genetic counselling and prenatal diagnostic testing to the family. The identification of novel variants is important to confirm phenotype variations of a syndrome.

## Background

Wiskott-Aldrich syndrome (WAS) occurs due to mutations in the *WAS* gene, located at Xp11.22-p11p23 [1, 2]. It is a rare X-linked recessive primary immunodeficiency disease (PIDD) originally described by the features of excess susceptibility to infections, eczema and microthrombocytopenia leading to bleeding disorders such as bloody diarrhea [1, 3, 4]. This is considered the most severe type, which often results in the development of autoimmunity, lymphoma or other malignancies. WAS almost exclusively affects males and the estimated incidence is less than 1 in 100,000 live births [5]. One of the hallmark characteristics of this disease is microthrombocytopenia, which is observed on a blood film and subsequently quantified using blood analysers [6]. In many cases, the diagnosis of WAS in first affected males is delayed because patients may not present with the classic signs and symptoms, which may intersect with thrombocytopenia causes [7–9]. In addition to classic WAS (50%) with total loss of function mutation, *WAS* mutations in are also associated with other disorders. Reduced WAS protein function mutations results inX-linked thrombocytopenia (XLT) (50%), while gain of function mutations cause the ultra-rare X-linked neutropenia (XLN) [7–9].

The *WAS* gene encodes the Wiskott-Aldrich syndrome protein (WASp), which consists of 502 amino acids and is a key regulator of actin cytoskeletal rearrangements [1]. Hematopoietic cells exclusively express WASp and the protein is implicated in a variety of functions such as immune synapse formation and cellular migration and hence impaired T and B cell function [10, 11]. Small platelets and congenital thrombocytopenia with a mean platelet volume of less than 5.0 fL in most affected persons are considered key to the diagnosis of WAS [10, 12]. We present the findings of an infant male patient with atypical features of WAS, suspected because of the clinical course and a relevant family history, although his platelets were initially normal in size and morphology. The demonstration of a novel mutation in *WAS* by exome sequencing provided a definitive diagnosis for appropriate treatment recommendations and counselling.

## Case presentation

At 16-weeks the infant was transferred to our hospital with pneumonia requiring assisted ventilation after prolonged hospitalization at a peripheral hospital. There he had presented with pneumonia and acute severe malnutrition, history of intermittent diarrhea, fevers and eczematoid skin rashes since 6 weeks of age. During the months of hospitalization, he developed Group B streptococcal bacteremia and pneumonia, cytomegalovirus (CMV) pneumonia and persistent viraemia, septic purulent knee, furuncles, buttock abscess, haemolytic anaemia, features of inflammatory bowel disease responsive to immune suppression, eosinophilic gastritis and multiple food allergies.

He developed chronic CMV viraemia requiring prolonged ganciclovir administration. The infant remained severely malnourished but never had signs of oedema. Ongoing diffuse erythrodermatous rashes appeared vasculitic (confirmed leukocytoclastic on histology) in areas, while other areas were more suggestive of eczema. The birth and perinatal history had been uncomplicated; the infant was exclusively breastfed and had tolerated primary vaccinations well including live BCG. A timeline for the presentation of symptoms of the index case is shown in Supplementary Fig. 1.

**Fig. 1:**
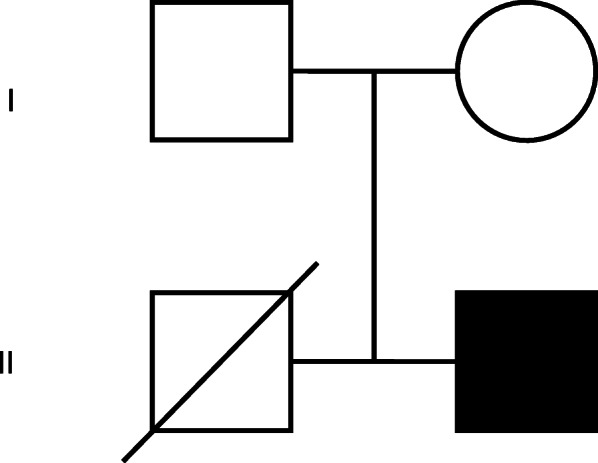
Multigeneration pedigree of the Wiskott Aldrich Syndrome family

There is no evidence for consanguinity of the parents. The family history was relevant however, for a 21-month older male sibling who had died at 8 months of age from infections. Perinatal history was normal, and the infant was breastfed. He had severe eczema since birth, failure to thrive and repeatedly documented low platelet counts. He had presented at 4 months to another hospital and was diagnosed with pneumocystis pneumonia and CMV infection. Platelet morphology and size were not recorded, but dysmorphic platelets with normal production of platelets were reported on a bone marrow aspirate done at the time of sepsis. Baseline immune investigations were normal. He demised with an undefined immunodeficiency diagnosis after unsuccessful evaluation for stem cell transplant (Fig. 1).

On systemic examination of the index patient at 16 weeks of age, there were no signs of dysmorphism, no enlarged lymph nodes nor hepatosplenomegaly. Human immuno- deficiency virus (HIV) polymerase chain reaction (PCR) of the infant and the mother’s HIV enzyme-linked immunosorbent assay (ELIZA) were negative. Initial full blood count (FBC) indicated normal white cell count and lymphocyte count, normocytic anaemia, eosinophilia and mild thrombocytopenia with a normal smear and no anomaly noted in platelet morphology. Platelet counts recovered on ganciclovir therapy for CMV infection. Investigations for immune deficit included serum immunoglobulins, expanded T cell subsets and T cell receptor excision circle (TREC) screening which were all normal, however elevated serum IgE levels were recorded. A summary of the all blood results is shown in Table 1. Following the WAS molecular diagnosis, electron microscopic exam of platelets confirmed microplatelets with reduced platelet diameter compared to adult controls. Gradually worsening thrombocytopaenia evolved over the first year of life but he did not suffer from any bleeding episodes. He was initially treated for undefined severe immunodeficiency and evaluated for stem cell transplantation, which the parents refused despite intensive counseling.

**Table 1:** Summary of blood results Jan 2020 – March 2015

	Normal	Jan 2020	April 2019	March 2018	Jan 2017	Feb 2016	Sept 2015	May 2015
Hb	10,7–14,7 g/dL	9,4 L	10,6	9,4	11,8	8,1 L	8,3 L	10,6
MCV	75–87 fl	71,3 L	72,8	71,6	83,8	69,4 L	79	76,8
WCC	6–16 × 10^9^/L	17,88	12,31	18,82 H	10,64	24,85 H	15,40	17,50
Plt	180–440 7,5 × 10^9^/L	133 L	81 L	77 L	67 L	113 L	84 L	234
MPV	7–11,4 fl	8,4	7	7,5	8,7	7,2	6,3 L	
IgG	5,04–14,65 g/L	11,98 H	13,63 H	15,12 H	23,17 H	11,53 H	12,52 H	
IgA	0,27–1,95 g/L	1,06	3,30 H	3,98 H	1,89	1,70 H	1,60 L	
IgM	0,24–2,10 g/L	0,26	0,56	< 0,16 L	0,30	0,34	0,39	
CMV VL			910 IU/ml/Log 3	909 IU/mL/ Log 3	8114 IU/mL/ Log 3,9	5704 IU/mL/ Log 3,8	24,454 IU/mL/Log 4,4	
EBV VL			5210 copies/mL/Log 3,7	22,300 copies/mL/log 4,3	1250 copies/mL/ log 3,1	IgM Neg		
CD3	2100–6200 cells/uL			6461 H		7808 H	11,232 H	
CD4	1300–3400 cells/uL			1151		878	775 L	
CD8	620–2000 cells/uL			4779 H		5856	10,457 H	
CD19	720–2600 cells/uL			620		878	646	
CD16/CD56	180–920 cells/uL			1593 H		781	1033 H	

The Health Research Ethics Committee of Stellenbosch University approved this study (study no. N13/05/075) and the ethical guidelines described in the “Declaration of Helsinki, 2013” were observed [13] . The parents of the affected infant child provided written informed consent which included permission for the genetic evaluation of the patient.. Venous blood for DNA extraction (using the Nucleon BACC3 Kit (Amersham Biosciences, Buckinghamshire, UK)) and whole exome sequencing (WES) was drawn from the affected child (1 ml) and both of his parents (5 ml).. An additional 5 ml of blood was drawn from the patient for full blood counts, platelet counts as well as electron microscopy.

### Exome capture and sequencing

The Ion AmpliSeq™ Exome RDY Kit as well as the Ion Xpress™ Barcode Adaptors 1–16 Kit (Life Technologies, Carlsbad, California, United States) were used for library preparation. Following this, the Ion PI™ Hi-Q™ Chef Kit followed by the Ion PI™ Chip Kit v3 was used to prepare the template DNA on the Ion Chef system. Sequencing reactions were conducted using the Ion Proton™ (Thermo Fisher, Carlsbad, California, United States) at Stellenbosch University’s Central Analytical Facility (CAF).

### Mapping, variant detection and annotation of variants

The Torrent Mapping Alignment Program (TMAP version 5.2.1) was used to align sequences to the human reference genome hg19 using the ion-analysis workflow available as part of the Torrent Suite (version 5.2.1). The variant caller (version 5.2.1.1) plugin on the Torrent suite was used for initial variant discovery and to subsequently generate a variant call format (VCF) file. Variant annotation was done using ANNOVAR [14]. To prioritize variants, a custom-designed, in-house method called TAPER™ was used [15].

### Sanger sequencing

A fragment of 275 base pairs containing the variant of interest was amplified by polymerase chain reaction from genomic DNA obtained from the patient and his parents. The following primers were designed: WASF- 5′ GTGGAGAGGAGATGGGAAAG − 3′ and WASR- 5′ CTGTGGATAGATGGATTGGGA-3′. Each amplicon was sequenced in a forward and reverse direction at Stellenbosch University’s Central Analytical Facility in Cape Town South Africa.

### Transmission Electron microscopy

Blood was centrifuged at 3000 rpm for 5 min. Plasma was subsequently removed and 2.5% Glutaraldehyde in 0.1 M buffer was added as the fixative. This was left to fix for 18 h. Platelets were removed, and 3% Osmium was added and left at room temperature for 1 h. The fixed sample was subsequently processed on a Leica Electron Microscopic Tissue Processor (Wetzlar, Germany). The following resins were added: 2% Uranyl acetate for 30 min, then 2X 70% alcohol 5 min each. 96% alcohol 5 min, 2% Uranyl nitrate 10 min, 100% alcohol for 10 min, 100% alcohol for 15 min; 100% alcohol for 20 min, 50/50 mixture of 100% alcohol and resin for 90 min, then both resins for 1 h each. The processed sample was then embossed in capsules filled with resin and placed in an incubator overnight at 65 °C. The sample was cut and stained with Toluidine Blue O for visualisation.

### Genetics findings

Uncertainty of diagnosis and suspected severe PIDD prompted urgent molecular investigation for our patient. Initial diagnoses included combined immunodeficiency or leaky severe combined immunodeficiency (SCID) and a bone marrow transplant was discussed but refused by the parents. The results obtained from the exome sequencing for the patient as well as his parents are presented in Supplementary Table 1. Data were filtered to prioritize rare and novel variants and a shortlist of three candidate variants was obtained (Supplementary Table 2). A novel hemizygous variant, *c. 397 G > A* in exon 4 of *WAS* (NM_000377.3)*,* was identified in the patient. This results in the substitution of glutamic acid to lysine at position 133 (E133K). Furthermore, applying the American College of Medical Genetics standards and guidelines to interpret sequence variants [16], this variant has been classed as likely pathogenic (One moderate and three supporting; PM2, PP2, PP3 and PP4).

**Fig. 2:**
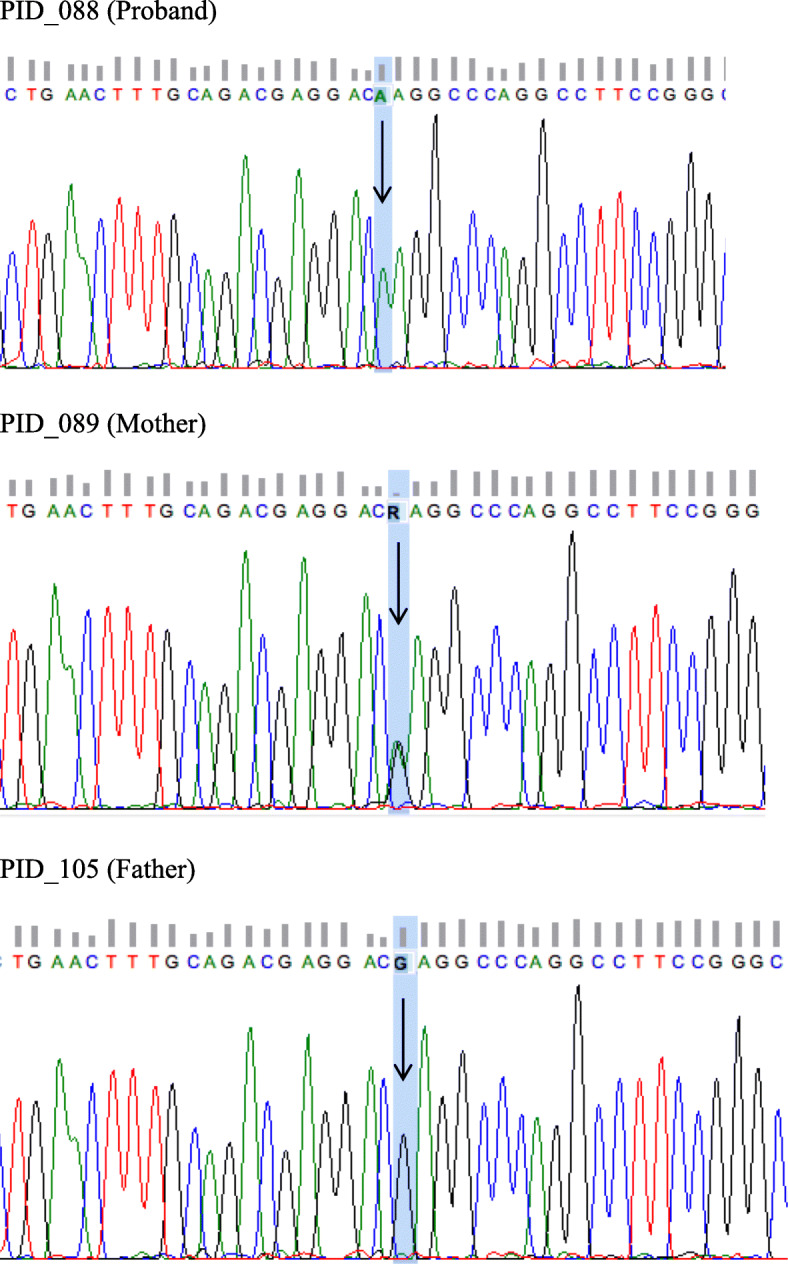
Sanger sequencing of proband (indicated by black arrow) and each of his unaffected parents. The mother of the proband is heterozygous for the *WAS c.397G > A* (p.133 E > K) while the proband is hemizygous for the mutation

**Fig. 3:**
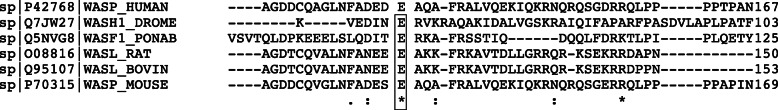
ClustalW multiple sequence alignment showing the position of the *c.397G > A* (p.133 E > K) mutation in *WAS*

The presence of the variant was validated by Sanger sequencing and the mutation details are summarized in Supplementary Table 3. It was determined that the mother is a heterozygous carrier of the variant, while it is completely absent in the father (Fig. 2). SIFT and PolyPhen2 was used for in silico predictions and indicated that *WAS c. 397 G > A* mutation found that it is damaging (Supplementary Table 3). Sequence alignment of multiple different species show that *WAS c. 397 G > A* is located at a position that is relatively well conserved across multiple species (Fig. 3). Further analysis of the protein identified that the variant is found in a WH1 domain, which is in control of the binding of proline-rich sequences in WASp. Moreover, analysis of the protein revealed that the amino acid substitution is from an amino acid with a negative charged to one which is positively charged and is therefore likely to affect protein folding.

### Microscopy findings

**Fig. 4:**
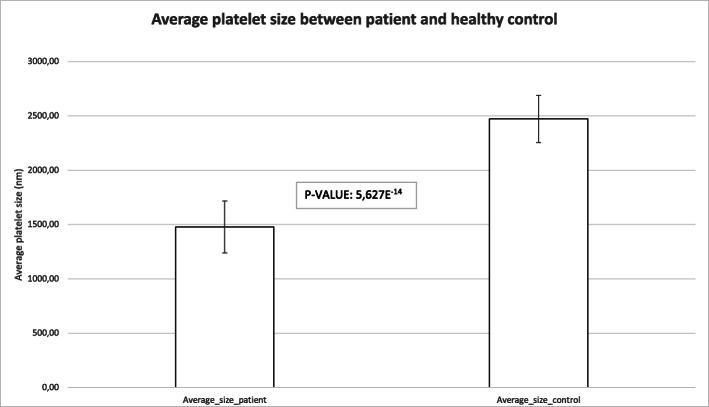
Mean platelet size of the patient versus the healthy control. The platelet size of the patient is significantly smaller than that of the healthy control, with a *P*-value of 5627e^− 14^

Following the identification of the E133K variant in *WAS*, platelet size and morphology were re-evaluated using transmission electron microscopy. This subsequent re-evaluation showed that the platelets of the patient are significantly smaller than those of a healthy aged-matched control individual (*p* = 5,627e^− 14^) (Fig. 4).

## Discussion and conclusion

To date, there are a total of three WAS protein mutation associated syndromes. These include classic WAS, congenital neutropenia without other WAS features and X-linked thrombocytopenia features [17, 18]. Here we summarise the case of a patient who presented with what was initially thought to be a combined or variant severe immunodeficiency. The diagnosis of WAS was considered in our patient because of his family history, despite initial normal platelet count and difficulty in establishing platelet volume and size measurements on routine analysis. Very few case descriptions of WAS patients with normal platelet size have been reported. In a study conducted by Patel et al. a young boy was the index case and presented with normal platelet size and thrombocytopenia. Given the clinical phenotype, WASp expression was evaluated using flow cytometry [19]. The index case had decreased expression of the protein when compared to a healthy individual. A c.826 A > T (K288X) mutation in exon 9 of the *WAS* gene was identified. WASp flow cytometric analysis was unfortunately not available for our patient. Using exome sequencing, we could identify a novel c.397 G > A (E133K) *WAS* mutation in exon 4*.*

The variant reported here was not found in any additional controls that were subjected to exome sequencing and it was determined that the variant falls in the WH1 domain. This domain is important as it binds to proline-rich sequences in the WASp interacting protein. Furthermore, it is anticipated that this domain has a vital role in numerous cellular events including signal transduction, protein folding and transport [18]. The identification of a novel variant in our patient in a hemizygous form, of which his mother is a carrier, will permit family counselling in the case of future pregnancies. Moreover, an early and accurate prenatal diagnosis by molecular testing such as exome sequencing will prevent potentially harmful procedures for example live vaccine administration and intramuscular injections, thereby preventing severe infection and internal bleeding. The patient currently receives home administered subcutaneous immunoglobulin and prophylactic antibiotics, eats a modified diet and continues in reasonable health, but is still failing to thrive.

Despite a positive family history of suspected PIDD in a deceased sibling, the definitive diagnosis in this child was complex. This was firstly due to repeated haematology laboratory reports of platelet clumping with use of conventional EDTA anticoagulation tubes. This led to suspicion of pseudothrombocytopenia due to antibody-induced platelet clumping or satellitism. Only once blood sampling in Sodium citrate anticoagulated tubes was applied routinely, were reliable reports of moderate thrombocytopaenia and reduced mean platelet volume obtained on automated analyzers from 1 year of age. Secondly the thrombocytopaenia reported with platelet clumping was further difficult to interpret against the clinical background with CMV infection and viral resistance. In addition to these complications, scanning electron microscopy is not routinely available to patients in South Africa, however it proved useful in this case because repeated platelet clumping on EDTA sampled blood. Eventual citrate anticoagulated blood samples by EM showed reduced MPV, whereas on automated processing this was only observed inconsistently. Furthermore micro thrombocytopaenia is not a reliable marker of WAS. In this setting, access to molecular diagnosis in a developing country, enabled us to make a confirmed PIDD diagnosis and counsel the mother who was in denial to accept such diagnosis.

The early diagnosis of WAS will also enable prompt referral for stem cell transplantation in severely affected patients. WAS is an extremely rare disorder with a broad clinical spectrum. Our data supports that of Baharin et al. [5] which suggests that a WAS diagnosis must be deliberated on for all male infants presenting with congenital thrombocytopenia irrespective of platelet size, with excess of infections and skin rashes and specifically in those where there is a recorded family history of unexplained male infant deaths.

## Supplementary information

**Additional file 1: Figure S1.** Timeline of symptoms presented by the index case.

**Additional file 2: Table S1.** Summary of exome sequencing data for the patient and his parents.

**Additional file 3: Table S2.** Shortlist of three candidate variants identified as plausible disease-causing variants.

**Additional file 4: Table S3.** Details of the candidate variant narrowed down using consecutive filters based on an autosomal recessive model of inheritance and low frequency.

## Data Availability

All whole exome sequencing data was aligned to human reference genome GCRh37 from the Genome Reference Consortium Human Build 37 (https://www.ncbi.nlm.nih.gov/assembly/GCF_000001405.13/). The reference sequence used for the validation of the E133K variant in WAS was obtained from NCBI Nucleotide using the accession number NM_000377.3. The variant reported in here is available in the Clinvar repository, [with accession ID: VCV000870492.1 (https://www.ncbi.nlm.nih.gov/clinvar/variation/870492/). The datasets generated during the current study are not publicly available because it is possible that individual privacy could be compromised and the participants did not provide consent to make the data public.

## Abbreviations

WAS: Wiskott-Aldrich Syndrome
WES: Whole exome sequencing
CMV: Cytomegalovirus
WASp: Wiskott-Aldrich Syndrome protein
XLN: X-linked neutropenia
XLT: X-linked thrombocytopenia
FBC: Full blood count
PCR: Polymerase Chain Reaction
PID: Primary immunodeficiency disease
CAF: Central Analytical Facility
SCID: Severe Combined Immunodeficiency
