# Supplementary figures and images for: Identification of a novel WAS mutation in a South African patient presenting with atypical Wiskott-Aldrich syndrome: a case report
Source: BMC Med Genet. 2020 Jun 5;21:124. doi: 10.1186/s12881-020-01054-6 (PMC7275612; doi:10.1186/s12881-020-01054-6)

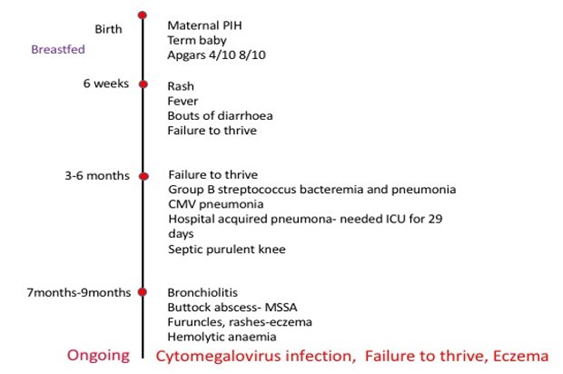

Supplement: Supplementary file 1 — Additional file 1: Figure S1. Timeline of symptoms presented by the index case. [file 12881_2020_1054_MOESM1_ESM.docx]
