# Supplementary material for: Identification of a novel WAS mutation in a South African patient presenting with atypical Wiskott-Aldrich syndrome: a case report
Source: BMC Med Genet. 2020 Jun 5;21:124. doi: 10.1186/s12881-020-01054-6 (PMC7275612; doi:10.1186/s12881-020-01054-6)
Supplement: Supplementary file 2 — Additional file 2: Table S1. Summary of exome sequencing data for the patient and his parents. [file 12881_2020_1054_MOESM2_ESM.docx]

**Table 1.** Summary of exome sequencing data for the patient and his parents.

|  | **Proband** | **Mother** | **Father** |
| --- | --- | --- | --- |
| **Total captured regions size** | 64 Mb | 64 Mb | 64 Mb |
| **% of captured regions with coverage >10** | 99.5 | 99.7 | 99.8 |
| **Average coverage of captured region (%)** | 98.9 | 98.6 | 99.1 |
| **Total number of SNPs** | 25,149 | 25,205 | 25,783 |
| **Total number of INDELs** | 559 | 564 | 548 |
| **N rare homozygous** | 33 | 21 | 26 |
| **N rare heterozygous** | 299 | 198 | 112 |
| **N X linked** | 15 | 7 | 11 |
| **N de novo events** | 75 | 61 | 44 |
