# Supplementary material for: Identification of a novel WAS mutation in a South African patient presenting with atypical Wiskott-Aldrich syndrome: a case report
Source: BMC Med Genet. 2020 Jun 5;21:124. doi: 10.1186/s12881-020-01054-6 (PMC7275612; doi:10.1186/s12881-020-01054-6)
Supplement: Supplementary file 3 — Additional file 3: Table S2. Shortlist of three candidate variants identified as plausible disease-causing variants. [file 12881_2020_1054_MOESM3_ESM.docx]

**Table 2.**  Shortlist of three candidate variants identified as plausible disease-causing variants.

| **Gene** | **SNV** | **dbSNP** | **1000 Genomes Project Frequency** | | | | | | **ExAC Genome Browser Frequency** | | | | | | | | | **ESP6500 Frequency** | | |
| --- | --- | --- | --- | --- | --- | --- | --- | --- | --- | --- | --- | --- | --- | --- | --- | --- | --- | --- | --- | --- |
|  |  |  | ALL | AFR | AMR | EAS | EUR | SAS | ALL | AFR | AMR | EAS | FIN | NFE | OTH | SAS | ALL | | AA | EA |
| *NRL* | G151R | None | 0.00 | 0.00 | 0.00 | 0.00 | 0.00 | 0.00 | 0.00 | 0.00 | 0.00 | 0.00 | 0.00 | 0.00 | 0.00 | 0.00 | 0.00 | | 0.00 | 0.00 |
| *WAS* | E133K | None | 0.00 | 0.00 | 0.00 | 0.00 | 0.00 | 0.00 | 0.00 | 0.00 | 0.00 | 0.00 | 0.00 | 0.00 | 0.00 | 0.00 | 0.00 | | 0.00 | 0.00 |
| *WNK1* | V724fs | rs35706572 | 0.00 | 0.00 | 0.00 | 0.00 | 0.00 | 0.00 | 0.00 | 0.00 | 0.00 | 0.00 | 0.00 | 0.00 | 0.00 | 0.00 | 0.00 | | 0.00 | 0.00 |

*NRL:* neutral retina leucine zipper; *WAS*: Wiskott-Aldrich Syndrome; *WNK1*: Serine/threonine-protein kinase WNK1; AFR: African; AMR: Ad Mixed American; EAS: East Asian; EUR: European; SAS: South Asian; FIN: Finnish; NFE: Non-Finnish European; OTH: Other; AA: African American; EA: European American.
