# Supplementary material for: Identification of a novel WAS mutation in a South African patient presenting with atypical Wiskott-Aldrich syndrome: a case report
Source: BMC Med Genet. 2020 Jun 5;21:124. doi: 10.1186/s12881-020-01054-6 (PMC7275612; doi:10.1186/s12881-020-01054-6)
Supplement: Supplementary file 4 — Additional file 4: Table S3. Details of the candidate variant narrowed down using consecutive filters based on an autosomal recessive model of inheritance and low frequency. [file 12881_2020_1054_MOESM4_ESM.docx]

**Table 3.** Details of the candidate variant narrowed down using consecutive filters based on an autosomal recessive model of inheritance and low frequency.

| **Chromosome** | Chr X |
| --- | --- |
| **Position** | 48544159 |
| **Gene name** | *WAS* |
| **RefSeq** | NM_000377 |
| **Reference sequence** | G |
| **Proband: number of reads with reference** | 0 |
| **Proband: alternative** | A |
| **Proband: number of reads with alternative** | 73 |
| **Mother: number of reads with reference** | 168 |
| **Mother: alternative** | A |
| **Mother: number of reads with alternative** | 168 |
| **Father: number of reads with reference** | 83 |
| **Father: alternative** | A |
| **Father: number of reads with alternative** | 0 |
| **Mutation type** | Missense |
| **Mutation: DNA (HGVS nomenclature _c.)** | 397 G>A |
| **Mutation: protein (HGVS nomenclature _p.)** | E133K |
| **Prediction < SIFT** | Damaging |
| **Prediction < PolyPhen-2** | Probably damaging |
| **Sanger verification** | Yes |
